# Hormonal and Sex-Specific Regulation of Key Players in Fibro-Calcific Aortic Valve Disease

**DOI:** 10.3390/ijms262110517

**Published:** 2025-10-29

**Authors:** Katherina Neussl, Sarah Werner, Holger Thiele, Florian Schlotter, Michael A. Borger, Petra Büttner, Julia Böttner

**Affiliations:** 1 Department of Cardiology, Heart Center Leipzig at University of Leipzig, 04289 Leipzig, Germany; 2 Department of Cardiology, University Medical Center, Johannes Gutenberg University Mainz, German Center for Cardiovascular Research—Partner Site Rhine-Main, 55131 Mainz, Germany; 3 Department of Cardiac Surgery, Heart Center Leipzig at University of Leipzig, 04289 Leipzig, Germany

**Keywords:** FCAVD, sex-specific, MGP, BMP2, fibronectin

## Abstract

Male sex and aging are risk factors for fibro-calcific aortic valve disease (FCAVD), indicating an understudied influence of sex hormones. Valvular interstitial cells (VICs) from female and male donors were isolated and exposed to pro-calcifying medium (PM), and the expression of matrix gla protein (MGP), fibronectin (FN1) and bone morphogenic protein 2 (BMP2) was analyzed. The effect of sex hormones on hydroxyapatite (HA) deposition by VICs was also analyzed. Exposure to PM increased MGP gene expression in male (*n* = 5, +5.8-fold, *p* = 0.031), and female VICs (*n* = 6, +4.9-fold, *p* = 0.004). In female VICs a +3.5-fold MGP increase accompanied the transition from the fibrotic to the calcific phase (*p* = 0.022 vs. males) while in male VICs the increase was delayed to the calcific phase. Female VICs upregulated FN1 (+1.8-fold, *p* = 0.003), while male VICs upregulated BMP2 (+3.7-fold, *p* = 0.05). 5α-dihydrotestosterone increased HA deposition +6.3-fold in male and +5.2-fold in female VICs (*p* ≤ 0.001 and *p* < 0.04, respectively). It further decreased BMP2 (*p* < 0.001) in male VICs and increased MGP in female VICs (*p* = 0.087). Female VICs decreased HA deposition when exposed to progesterone (−2.4-fold, *p* = 0.037 vs. PM) and estrogen (−2.0-fold, *p* = 0.072). In summary, VICs show donor-sex-specific gene expression which is modifiable by 5α-dihydrotestosterone. This needs to be considered when designing in vitro regulatory studies.

## 1. Introduction

Fibro-calcific aortic valve disease (FCAVD) is highly prevalent in the aging populations of high-income countries [1,2]. FCAVD is characterized by fibrous tissue accumulation with subsequent calcific mineral deposition within the aortic valve (AV) leaflets, resulting in valvular dysfunction. Stimuli such as inflammation, mechanical stress and oxidative stress activate resident valvular interstitial cells (VICs) towards myofibroblastic or osteoblastic differentiation, leading to remodeling of the extracellular matrix (ECM), fibrosis and calcification [3], and ultimately AV stenosis. Despite the increasing knowledge of FCAVD pathomechanisms, no pharmacological intervention is yet available, leaving AV replacement the only therapeutic option.

Sex is well known to play a substantial role in the development and progression of FCAVD, as male sex is recognized as a risk factor for FCAVD [4]. At the same degree of stenosis, male patients present with higher degrees of calcification, while female patients present more non-calcified fibrotic ECM expansion [5,6]. Research on the underlying pathomechanisms of this sexual dimorphism is crucial for the development of targeted sex-specific therapies. Therefore, this study aimed to characterize sex-specific patterns of key players involved in fibrotic and calcific processes in FCAVD in vitro utilizing a pro-calcifying cell culture medium (PM). Recently, VICs treated with PM were found to show a biphasic temporal profile in an impedance-based cell monitoring system. Impedance declined within the first eight days of exposure to PM, reflecting pro-fibrotic cell activation and inhibition of cell proliferation. The cellular impedance then increased until day 20, coinciding with advanced calcification equivalent to an advanced disease stage [7]. These experimental conditions were adopted in this study and gene expression or protein abundance was determined at day 7 (=fibrotic phase) and at day 20 (=calcific phase) and in cells exposed to control medium (CM).

Bone morphogenetic protein 2 (BMP2) is a member of the transforming growth factor beta (TGF-β) family and promotes the osteogenic transformation and mineralization of VICs via Wnt/β-catenin signaling in osteoblasts and chondrocytes, modulating cell proliferation and differentiation [8]. The transcription factor is primarily regulated at the protein level, so protein abundance is a reliable indicator of its cellular activity in vitro [9]. Importantly, BMP is regulated by matrix gla protein (MGP), a vitamin K-dependent protein that inhibits vascular calcification by two major mechanisms. MGP actively binds hydroxyapatite (HA) crystals and thus prevents crystal growth [10] and indirectly controls calcification by attenuating BMP2 signaling [11]. As MGP is poorly soluble and prone to aggregation, protein analysis is challenging and thus gene expression analysis is preferred to characterize MGP [12].

The glycoprotein fibronectin (FN1) is involved in cell–cell interactions [13] and valvular homeostasis [14] by promoting pro-osteoblastic differentiation and collagen production [15]. As FN1 is rapidly secreted and accumulates in the ECM, gene expression levels are characterized in pathomechanistic studies [16].

We hypothesized that BMP2 as a pro-osteogenic molecule and MGP with its potential cardioprotective activity might be regulated differently in male and female FCAVD patients. Further, we hypothesized that pronounced fibrosis in female VICs might be reflected by a higher abundance of FN1.

While ambiguous evidence exists regarding the impact of sex hormones on promoting or protecting against calcific nodule formation, multiple studies have elucidated a protective role of androgens in FCAVD [17,18,19]. Physiological levels of 5α-dihydrotestosterone (5α-DHT) were found to prevent vascular calcification by reducing the signaling of TGF-β [20], sustaining the activity of endothelial nitric oxide synthase [21] and suppressing local inflammatory signaling in vitro [22]. Further, an inverse association between low 5α-DHT levels and pronounced vascular calcification in older men with stable coronary artery disease was demonstrated [17]. In addition, men with low testosterone levels were found to be more prone to early onset atherosclerosis [23]. Estrogens suppress calcification by repressing RANKL and NF-κB signaling [24,25], or by regulating NADPH oxidase activity [26] and p53 [27]. Importantly, estrogen induced MGP gene expression [25,28], increased nitric oxide synthase expression and -activity and led to the upregulation of antioxidant enzymes in vitro [29,30,31]. The role of progesterone in FCAVD is unknown.

Although there is an awareness of sex-specificity in FCAVD, most ex vivo studies do not consider donor sex and hormonal status as biological variables.

This study assessed the impact of the donor sex of VICs that are commonly used as FCAVD in vitro model. Additionally, the effect of sex hormone stimulation on in vitro calcification of VICs from of both sexes was analyzed.

As proof of concept, the sex-specific expression patterns of MGP, BMP2 and FN1 in AVs and in VICs exposed to a pro-calcifying environment were investigated, and exemplarily the effect of 5α-DHT supplementation was tested.

## 2. Results

VICs from thirteen male patients and fourteen female patients with comparable age (years) ((median [interquartile range]) males: 69 [63, 76], females: 71 [65, 73], *p* = 0.670), body mass index (kg/m^2^) (males: 26.3 [25.3, 33.4]; females: 29.2 [24.5, 35.1], *p* = 0.492) and left ventricular ejection fraction (%) (males: 56 [45, 62]; females: 62 [43, 64], *p* = 0.626) were analyzed. All patients except one underwent AV replacement due to severe AV stenosis. One male patient suffered from AV regurgitation. Five male and five female patients had diabetes mellitus type 2. One male and four female donors had chronic kidney disease.

### 2.1. Impact of Donor Sex and Sex Hormones on In Vitro Calcification

**Figure 1:**
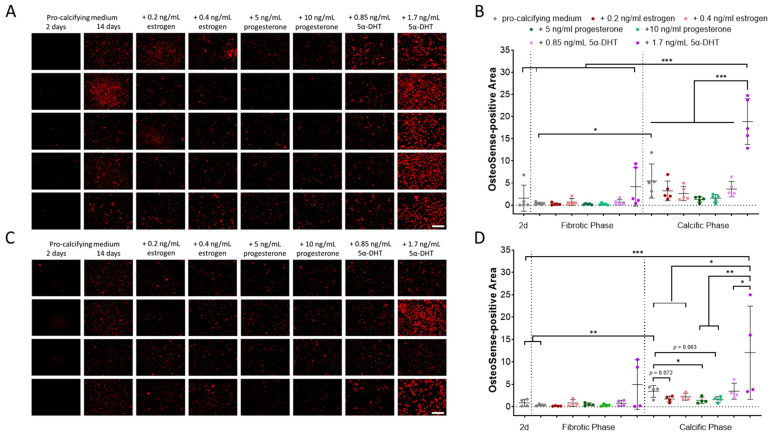
Sex-specific hydroxyapatite formation by VICs exposed to pro-calcifying medium and sex hormones. Near-infrared molecular imaging and quantification of hydroxyapatite formation of (**A**,**B**) five male and (**C**,**D**) four female VIC cultures exposed to pro-calcifying medium are shown exemplarily at day 2 and 14. Additional sex hormone supplementation after 14 days is displayed. d: days. 5α-DHT: 5-alpha-dihydrotestosterone. Scale bar: 200 µm. Mean ± SD. * *p* < 0.05, ** *p* < 0.01, *** *p* < 0.001.

Calcification was monitored using the HA-specific molecular probe OsteoSense. PM-stimulated HA deposition by male and female VICs increased over time without sex-specific differences (Figure 1A,C). The OsteoSense-positive area was increased +3.4-fold in male VICs (*p* = 0.037) and +3.9-fold in female VICs (*p* = 0.007) after 14 days in PM compared to day 2 (Figure 1B,D). When sex hormones were added to PM, HA formation after 14 days was affected. Supplementation with 5α-DHT led to a +6.3-fold increase in HA deposition in male VICs (*p* ≤ 0.001 vs. all other experimental groups) and a +5.2-fold increase in female VICs (*p* < 0.04 vs. all other experimental groups). In female VICs HA deposition was significantly suppressed by progesterone (−2.4-fold, *p* = 0.037 vs. PM) and by trend also by estrogen supplementation (−2.0-fold; *p* = 0.072 vs. PM). No effects of estrogen and progesterone on HA deposition by male VICs were observed.

### 2.2. MGP, FN1 and BMP2 Expression in Relation to Donor Sex and After Exposure to 5α-DHT

Gene expression of MGP, FN1 and BMP2 was analyzed in AVs subdivided into mildly diseased, fibrotic and calcified areas (Figure S1). Gene expression of FN1 and BMP2 was comparable in all areas and independent of donor sex or analyzed area. However, MGP gene expression by trend was higher by 1.6-fold in calcified areas of AVs from female donors compared to the mildly diseased areas (*p* = 0.099).

**Figure 2:**
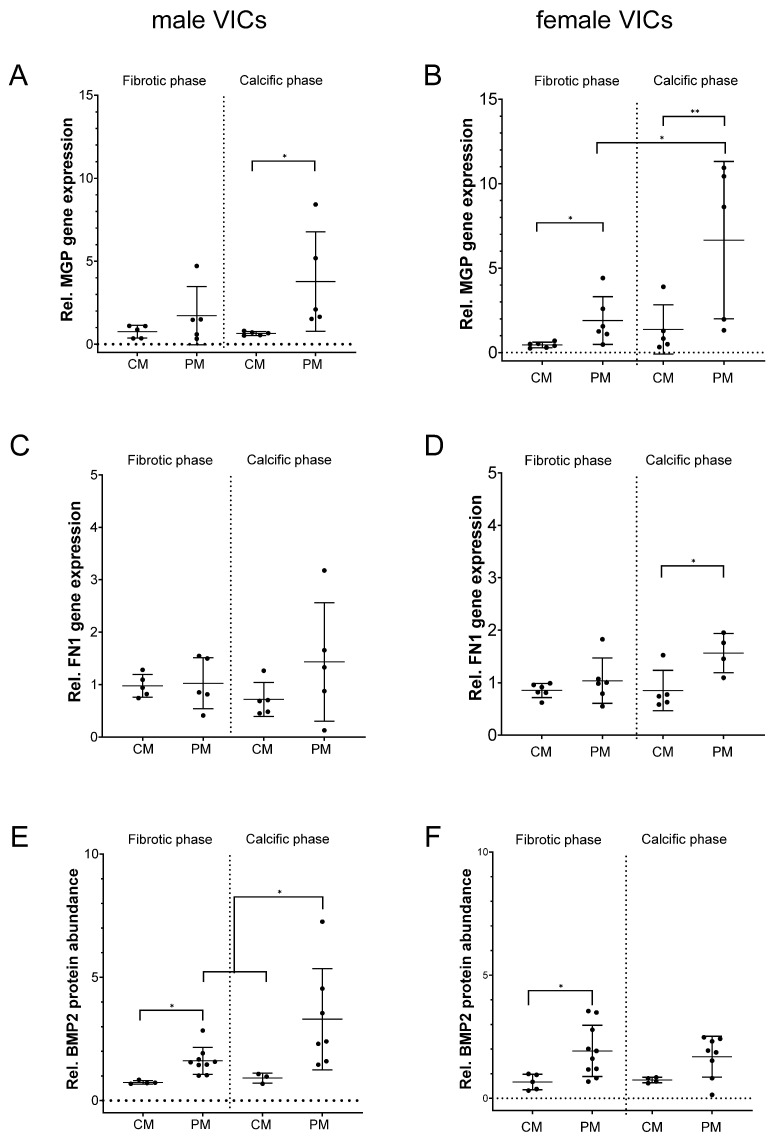
Sex-specific gene expression and protein abundance in VICs during fibrotic and calcific remodeling in pro-calcifying medium. Relative MGP gene expression (**A**,**B**), relative FN1 gene expression (**C**,**D**) and relative BMP2 protein abundance (**E**,**F**) in male (**A**,**C**,**E**) and female VICs (**B**,**D**,**F**) in control medium (CM) and in pro-calcifying medium (PM) during the fibrotic and calcific phases. Mean ± SD. *n* ≥ 3. * *p* < 0.05; ** *p* < 0.01.

MGP, FN1 and BMP2 expression was also analyzed in vitro in VICs exposed to pro-calcifying conditions (Figure 2).

After 20 days of VIC exposure to PM, MGP gene expression was enhanced +5.8-fold in male (*p* = 0.031) and +4.9-fold in female VICs (*p* = 0.004) compared to CM. However, in female VICs a pronounced +3.5-fold increase (*p* = 0.022) in MGP had occurred already during the transition from the fibrotic to the calcific phase at day 7, while this was not visible in male VICs where a high inter-individual variation was observed (+2.2-fold, *p* = 0.983) (Figure 2A,B).

FN1 gene expression was significantly increased by +1.8-fold (*p* = 0.027 vs. CM) in VICs from female donors (Figure 2D), while no changes were observed in VICs from male donors (*p* = 0.240 vs. CM, Figure 2C). During the fibrotic phase BMP2 protein was significantly increased in male (+2.2-fold, *p* = 0.020 vs. CM) and female VICs (+3.0-fold, *p* = 0.04 vs. CM). Interestingly, in the calcific phase, BMP2 further increased in VICs from male donors (+3.7-fold, *p* = 0.050 vs. CM) (Figure 2E), while a less pronounced trend towards an increase (+2.3-fold, *p* = 0.051 vs. CM) was observed in VICs from female donors (Figure 2F).

**Figure 3:**
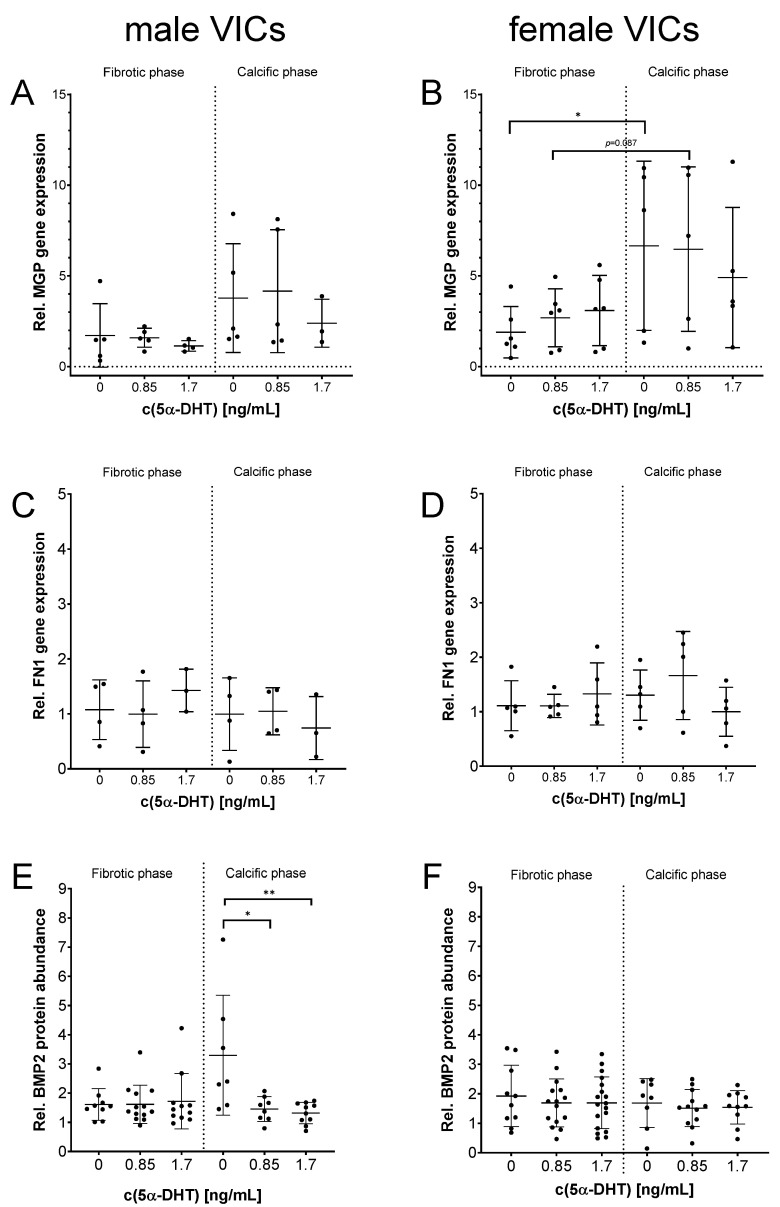
Impact of 5α-dihydrotestosterone (5α-DHT) on the gene expression of MGP (**A**,**B**) and FN1 (**C**,**D**) and the protein abundance of BMP2 (**E**,**F**) in the fibrotic and calcific phases in male (**A**,**C**,**E**) and female (**B**,**D**,**E**) VICs. Mean ± SD. n ≥ 3. * *p* < 0.05, ** *p* < 0.01.

5α-DHT had a pronounced impact on the HA-specific calcification of all VICs (Figure 1). Thus, the impact of 5α-DHT on MGP and FN1 gene expression and BMP2 protein abundance was analyzed (Figure 3).

By trend, an increase in MGP gene expression after exposure to 0.85 ng/mL 5α-DHT in the calcific phase in female VICs was observed (Figure 3B). FN1 gene expression was not affected (Figure 3C,D). Interestingly, stimulation of male VICs with 0.85 ng/mL and 1.7 ng/mL 5α-DHT prevented the PM-driven enhancement of BMP2 in the calcific phase (*p* < 0.001, Figure 3E). BMP2 protein expression in VICs from female donors was not changed (Figure 3F).

## 3. Discussion

Patients with chronic kidney disease (CKD) show accelerated heart valve calcification [32] as the hyperphosphatemic environment in these patients induces osteoblastic differentiation in VICs and finally results in the formation of calcific nodules [33]. The hyperphosphatemic PM is used to recapitulate these processes in vitro. VICs are triggered by PM towards fibrosis, osteogenesis and calcification, which is characterized by HA deposition in vitro. In vitro, these cellular alterations are reflected by a biphasic response, representing an initial fibrotic activation and differentiation until day 8 of PM exposure (fibrotic phase), followed by progressing calcification until day 20 (calcific phase) [7].

Using this cell model, it is demonstrated here that MGP and FN1 gene expression as well as BMP2 protein expression increase during the calcific phase. This is in line with results from CKD patients where high BMP2 serum levels [34] and even higher levels of the inactive form of MGP were observed, which were associated with increased vascular calcification [35]. While by trend MGP gene expression was increased in calcific areas of AVs from female donors, this observation was validated and was further enhanced in vitro by isolated female VICs exposed to PM.

In conclusion, exposure of VICs to PM in vitro induces FCAVD-specific features, which underpins the suitability of this in vitro FCAVD model.

Clinical observations suggest sex-specific FCAVD characteristics wherein men present with more calcified valves and females present with more fibrotic valves [36,37].

BMPs, TGF-β superfamily members, are discussed as being the most pivotal anabolic elements in the process of osteogenesis within the human body [38]. BMP2 has repeatedly been associated with calcification of the AV and the vascular system as it enhances osteoblastic differentiation of VICs and vascular smooth muscle cells [39,40,41]. Subsequent studies have shown that BMP2 is upregulated solely in male VICs upon exposure to pro-calcifying stimuli, such as IFN-γ [42] or aldosterone [43]. Here, VIC exposure to PM, an in vitro surrogate of a hyperphosphatemic environment, had the same effect on male VICs only, providing a possible explanation for the more pronounced calcific phenotype in male FCAVD patients.

MGP was shown to be an inhibitor of vascular calcification in vivo and in vitro [10,40]. It prevents and may even reverse calcification by removing calcium phosphate particles from the ECM or even from the arterial wall [44,45,46]. Furthermore, it regulates BMP2 activity by blocking its interaction with the specific transmembrane receptors BMPR 1 and 2. MGP was thus supposed to prevent the transformation of vascular smooth muscle cells into osteoblasts [47,48]. Although MGP typically inhibits bone formation, it may also stimulate osteo-induction, depending on the relative ratio of MGP to BMP2 [49].

In this study, MGP was upregulated in both male and female VICs during the calcific phase. Interestingly, in vitro only female VICs exhibited a considerable increase in MGP expression already during the fibrotic phase, which proceeded during the transition from the fibrotic to the calcific phase. This finding is in line with the observed upregulation of MGP gene expression in the calcific areas of AVs from the female donors.

This increase in MGP gene expression in female VICs may impede BMP2 expression in the form of a compensatory mechanism, and may thus explain the reduced calcification that is observed in female FCAVD patients [5,36].

In female VICs exposed to PM, FN1 gene expression was elevated in the calcific phase, while in male VICs FN1 gene expression remained comparable to the control. This sex-specific regulation of FN1 has been unknown so far. However, it was observed in a mouse model of AV stenosis that, although an upregulation of the pro-fibrotic genes TNF-α and TGF-β was observed in both sexes, the extent of fibrosis was lower in male mice and was subsequently replaced by calcification [50]. Likewise, stenotic valves from female patients have higher collagen content, representing pronounced fibrotic remodeling, than those from males [36]. Conclusively, while the fibrotic phenotype is regarded as a preceding condition in FCAVD [5], which progresses to calcification in men [37,42], female-specific suppression of pro-osteogenic genes and prolonged expression of pro-fibrotic genes like FN1 may preserve this phenotype.

Male sex and aging are important FCAVD risk factors pointing towards a potential involvement of altered sex hormone levels. Similarly, the postmenopausal drop in estrogens is a relevant risk factor for manifestation and progression of FCAVD in female patients [51].

This study supports the protective role of estrogen and progesterone in FCAVD as these hormones were found to suppress PM-mediated HA deposition in vitro. Estrogen is well documented to induce MGP gene expression in estrogen receptor-positive cells [25,28]. Additionally, MGP binds HA and thus inhibits HA crystal growth [10]. It may be hypothesized that estrogen-mediated MGP upregulation represents a protective mechanism against HA formation in women. Estrogen supplementation or hormone replacement therapy during menopause might have beneficial effects on onset and progression of female valve calcification [51]. While protective effects of estrogen are well established, potential effects of progesterone are understudied in the context of FCAVD. Here, a progesterone-mediated repression of HA formation, which was more pronounced in female VICs, indicates a beneficial impact on FCAVD.

Among the analyzed sex hormones 5α-DHT had the strongest impact on HA crystal growth, whereas the impact on gene and protein expression has been studied exemplarily in more detail. The role of 5α-DHT and its effect on cardiovascular diseases have been discussed controversially. While low 5α-DHT levels increase the risk for atherosclerosis, coronary artery disease and cardiovascular events [19], young athletes with supraphysiological 5α-DHT levels also show noticeably higher levels of coronary artery disease [52]. 5α-DHT levels start to decline after the third decade of life and are bisected after the sixth decade [18]. Since this study included VICs from males with an average age of 69 years, an age-related significant decline in serum-5α-DHT can be assumed. Importantly, supplementation with 5α-DHT in vitro reversed PM-driven BMP2 protein upregulation specifically in male VICs.

However, this study also showed a significant increase in HA formation in PM-treated VICs supplemented with 5α-DHT.

These findings indicate that although 5α-DHT may act anti-osteogenic by downregulating BMP2, 5α-DHT also mediates pro-osteogenic mechanisms, indicated by the reinforced HA formation. It is assumed that 5α-DHT executes its pro-osteogenic mechanisms via BMP2-independent signaling pathways. For example, Son and colleagues have shown anti-calcification effects of testosterone and 5α-DHT via androgen receptor-activated survival pathways in human aortic vascular smooth muscle cells [53]. The way by which HA deposition is increased here remains elusive, but BMP2-independent activation of β-Catenin/Wnt signaling or the activation of other pro-calcific pathways [54] should be analyzed.

Consequentially, we assume that augmenting 5α-DHT levels in male patients should be regarded as a sex-specific approach for FCAVD treatment, but we urge that further research has to be conducted as the molecular implications are complex.

## 4. Materials and Methods

### 4.1. Aortic Valves and Isolation and Cultivation of Human Valvular Interstitial Cells

AVs from patients who underwent surgical AV replacement at the Department of Cardiac Surgery at the Heart Center Leipzig were used for this study. The study was approved by the local Ethical Committee at the Medical Faculty, University Leipzig (registration number 128/19-ek), and all patients provided written informed consent, in accordance with the Declaration of Helsinki. AVs from eight male and female donors, respectively, were dissected into mildly diseased, fibrotic and calcific areas. Samples were snap-frozen in liquid nitrogen immediately and pulverized using a liquid nitrogen-precooled cell crusher.

AVs from another five male and six female donors were subjected to VIC isolation as described previously [7]. In brief, AVs were minced, enzymatically digested using collagenase for four hours, passed through a 40 µm cell strainer and cultivated in high-glucose DMEM supplemented with 10% fetal bovine serum and 1% penicillin/streptomycin. Experiments were conducted in the passages three to four when the cells were at approximately 100% confluency. Control cultures were grown in high-glucose DMEM (+5% FBS + 1% penicillin/streptomycin), referred to as CM. Calcification was induced using PM representing CM supplemented with 2 mM sodium dihydrogen phosphate (NaH_2_PO_4_, pH 7.4) and 50 µg/mL L-ascorbic acid (Merck, Darmstadt, Germany). To study the effect of sex hormones, PM was supplemented with 0.2 or 0.4 ng/mL 17-estradiol [55], 5 or 10 ng/mL progesterone [55] and 0.85 or 1.7 ng/mL 5α-DHT [56], purchased from Selleck Chemicals (Houston, TX, USA). VICs were cultivated over a period of 20 days, and were harvested in QIAzol Lysis reagent or RIPA buffer and stored at −80 °C until further use.

### 4.2. Near-Infrared Molecular Staining and Quantification of PM-Induced HA Deposition

HA deposition by VICs was visualized by adding a 2 nmol staining solution of the HA-specific near-infrared, bisphosphonate-based molecular probe (IVISense Osteo 680 EX, Perkin Elmer, Waltham, MA, USA) to VIC cell cultures from five male and four female donors for 24 h. Visualization was performed at 680 nm using a fluorescence microscope (Keyence, BZ-X800, Itasca, IL, USA). The OsteoSense-positive area was quantified using ImageJ (version 1.54p) [57].

### 4.3. Quantitative Real-Time Polymerase Chain Reaction (qRT-PCR)

All samples were passed through QIAShredder columns before RNA isolation using the RNeasy Kit (Qiagen, Hilden, Germany) following the manufacturer’s instructions. The Omniscript RT Kit with oligo-dTPrimer (Qiagen, Hilden, Germany) was used for reverse transcription of 100 ng RNA according to the manufacturer’s protocol. Takyon NoRox Sybr Mastermix Blue (Eurogentec, Lüttich, Belgium) and a BioRad CFX system (BioRad, Hercules, TX, USA) were used. Exon-spanning primers with an annealing temperature of 60 °C were designed (sequences in Table 1).

Standard curves were used to calculate copy numbers and reaction efficiency. Measurements were performed in triplicate. Hypoxanthine phosphoribosyltransferase 1 (HPRT1) was used for expression normalization of the target genes. Samples not detected until a cycle threshold of 37 were considered as not expressed and were excluded from the analysis. Specimens with a technical replicate standard deviation > 0.3 were excluded.

### 4.4. Western Blot

Protein samples were harvested in RIPA buffer, ultrasonicated, separated on 12% SDS–polyacrylamide gels and subsequently transferred to a polyvinylidene fluoride membrane. Anti-BMP2 (Abcam, Cambridge, UK, ab284387, dilution 1:1000, 38 kDa), anti-α-tubulin (Abcam, ab7291, dilution 1:10,000, 50 kDa), secondary POD-labeled antibodies (Sigma Aldrich, St. Louis, MO, USA, A0545, dilution 1:10,000 and A9044-2ML, dilution 1:5000) and enzymatic chemiluminescence detection (Super Signal West Pico, Thermo Fisher Scientific Inc., Bonn, Germany) were used. Protein amounts of BMP2 were normalized to those of α-tubulin.

### 4.5. Statistical Analysis

IBM SPSS Statistics (Version 24) was used for statistical analyses. The data were tested for normal distribution using the Shapiro–Wilk test and are presented as mean ± standard deviation (SD). The number of replicates was denoted as “*n*” and refers to the number of donors for each condition in each dataset. All measurements were performed using biological replicates. Raw data from Western blot and qRT-PCR were median-normalized to correct for batch effects when significant plate-to-plate or blot-to-blot median differences were observed. Group differences were analyzed using one-way analysis of variance (Welch-ANOVA) or Student’s *t*-test. Time traces were analyzed by two-way repeated measures ANOVA. Tukey’s post hoc test was used. *p*-values < 0.05 were considered statistically significant. *p*-values < 0.1 were considered as a trend.

## 5. Conclusions

VICs exposed to pro-calcifying conditions in vitro show increased expression levels of BMP2, FN1 and MGP, which are all important key molecules in FCAVD. Interestingly, these regulations are sex-specific. VICs from males display strong upregulation of BMP2, whereas 5α-DHT can counteract this effect, suggesting a protective role in FCAVD progression in vitro. VICs from females show a more pronounced increase in MGP, which is a natural antagonist to BMP2 signaling.

In general, it is highly recommended to consider the donor sex when VICs are used for in vitro studies on FCAVD pathomechanisms. In addition, the first findings from this study may be used for hypothesis generation and future experimental design in order to improve the understanding of sex-specific differences in FCAVD and to emphasize the need for targeted therapies, which take the sex and hormone status of patients into account.

### Limitations

Due to the limited number of participants and the observed pronounced inter-individual variability, subtle effects might not have been detected.

Further, due to technical limitations in estimating MGP gene expression and BMP2 protein abundance, we were not able to calculate an in vitro MGP/BMP2 ratio.

Due to the limited proliferation capacity of isolated VICs and the restriction of experiments to passages three to four, expression levels of sex hormone receptors could not be determined in the present study.

## Figures and Tables

**Table 1:** Primers used for quantitative real-time PCR.

Gene	Forward Primer	Reverse Primer
*HPRT1*	CTCATGGACTGATTATGGACAGGAC	GCAGGTCAGCAAAGAACTTATAGCC
*MGP*	CCCTATTGAGCTCGTGGACA	ACCTTCATATCCCCTCAGCAGA
*FN1*	TCGTGCTTTGACCCCTACAC	TTCCCAAGACATGTGCAGCT

## Data Availability

All data supporting the findings of this study are available within the paper. Primer sequences are provided in Table 1.

## Supplementary Materials

The following supporting information can be downloaded at: https://www.mdpi.com/article/10.3390/ijms262110517/s1.
