# Supplementary material for: Hormonal and Sex-Specific Regulation of Key Players in Fibro-Calcific Aortic Valve Disease
Source: Int J Mol Sci. 2025 Oct 29;26(21):10517. doi: 10.3390/ijms262110517 (PMC12607358; doi:10.3390/ijms262110517)
Supplement: Supplementary file 1 [file ijms-26-10517-s001.zip › ijms-3867010-supplementary.pdf]

Supplementary Information

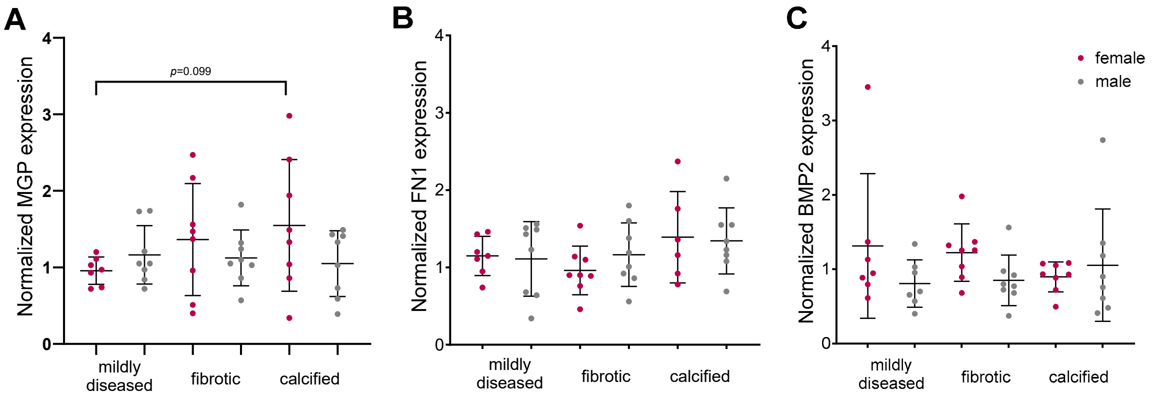

Supplementary Figure S1: Normalized gene expression of MGP, FN1 and BMP2 in stenotic AVs from female and male patients. Gene expression was normalized to HPRT gene expression. *n*=8 samples/donor sex with 6-8 samples per area. Mean ± SD. Unpaired t-test.
